# Identification and Functional Characterization of *MdNRT1.1* in Nitrogen Utilization and Abiotic Stress Tolerance in *Malus domestica*

**DOI:** 10.3390/ijms24119291

**Published:** 2023-05-26

**Authors:** Guodong Liu, Lin Rui, Yuying Yang, Ranxin Liu, Hongliang Li, Fan Ye, Chunxiang You, Shuai Zhang

**Affiliations:** 1 National Key Laboratory of Crop Biology, National Research Center for Apple Engineering and Technology, College of Horticulture Science and Engineering, Shandong Agricultural University, Tai’an 271018, China; 13658605600@163.com (G.L.); chrisone2020@163.com (L.R.); 17863807915@163.com (Y.Y.); lr17686260513@163.com (R.L.); li12292819@163.com (H.L.); 15386677267@139.com (F.Y.); 2 Key Laboratory of Agricultural Film Application of Ministry of Agriculture and Rural Affairs, College of Chemistry and Material Science, Shandong Agricultural University, Tai’an 271018, China

**Keywords:** MdNRT1.1, nitrate uptake, abiotic stress tolerance, hormonal response

## Abstract

Nitrate is one of the main sources of nitrogen for plant growth. Nitrate transporters (NRTs) participate in nitrate uptake and transport, and they are involved in abiotic stress tolerance. Previous studies have shown that NRT1.1 has a dual role in nitrate uptake and utilization; however, little is known about the function of MdNRT1.1 in regulating apple growth and nitrate uptake. In this study, apple *MdNRT1.1*, a homolog of *Arabidopsis NRT1.1*, was cloned and functionally identified. Nitrate treatment induced an increased transcript level of *MdNRT1.1*, and overexpression of *MdNRT1.1* promoted root development and nitrogen utilization. Ectopic expression of *MdNRT1.1* in *Arabidopsis* repressed tolerance to drought, salt, and ABA stresses. Overall, this study identified a nitrate transporter, MdNRT1.1, in apples and revealed how MdNRT1.1 regulates nitrate utilization and abiotic stress tolerance.

## 1. Introduction

As a major nutrient element, nitrogen (N) plays a key role in metabolism in various organisms [1,2,3,4,5,6,7,8]. Nitrogen constitutes around 4% of the total dry weight of plants. Nitrate (NO_3_^−^) is an essential source of nitrogen for the majority of agriculturally cultivated plants [9]. The use of a high quantity of nitrogen fertilizer increases planting expenses and diminishes the soil’s natural fertility [10,11,12,13,14,15]. Overuse of nitrogen fertilizer has therefore resulted in nitrogen pollution, which is now acknowledged as a danger to the sustainability of the environment [15,16]. Therefore, improving the effective uptake of nitrate by plants is a key goal for breeders worldwide [1,2,3,9,17,18,19,20,21,22,23,24,25,26,27,28].

Plants mainly rely on transmembrane proteins to absorb nitrate from the outside; these proteins regulate nitrate transport in plants’ cells, tissues, and organs. Nitrate transport is mainly regulated by four major protein families: the NITRATE TRANSPORTER 1 (NRT1)/PEPTIDE TRANSPORTER (PTR) family (NPF), NITRATE TRANSPORTER 2 (NRT2), CHLORIDE CHANNEL (CLC) family, and SLOWLY ACTIVATING ANION CHANNEL [9]. Among them, *NRT1.1* has numerous roles and has been widely studied. *NRT1.1* predominates in root nitrate uptake under nitrogen-filled conditions. Once the plant root cells absorb the nitrate, it is assimilated or stored in the root and transported to all parts of the plant body [20]. In *Arabidopsis*, *AtNRT1.1* was cloned and is an indispensable nitrate transporter that exhibits a characteristic dual affinity, making possible the use of *AtNRT1.1* to search for homologous genes of NRT1.1 in other species [29,30,31].

Additional to nitrate transport, NRT1.1 is also widely involved in the regulation of plant responses to various abiotic stresses, such as drought and salt stress. Abiotic stresses, such as drought and salt stress, affect the survival of 40% of the world’s crops, particularly by affecting nitrate uptake by plant roots [32,33,34]. In addition, as a special stress hormone, ABA biosynthesis is closely related to nitrate supply and allows plants to minimize the effects of adverse conditions in order to survive [32,35]. Researchers have identified numerous plant mechanisms for adapting to drought tolerance, and they have found that *NRT1.1*-mediated nitrate transport is also involved in the drought response [35]. In *Arabidopsis*, *AtNRT1.1* is abundantly expressed in the guard cells of leaves, and this expression increases the concentration of nitrate to induce the guard cells to be depolarized to promote stomata opening, suggesting that *NRT1.1*-mediated nitrate signaling may play a role in plant health under arid conditions [35,36]. *NRT1.1* was also found to be involved in the salt stress response [37]. Since nitrate enhances Na^+^ absorption in roots and Na^+^ buildup in stems, one or more nitrate transporters may govern plant Na^+^ transport [37,38]. Although the Na^+^ accumulation of *NRT1.1* mutants was significantly lower than that of wild-type plants, this difference disappeared after nitrate removal. This finding suggested that *NRT1.1* may regulate NO_3_^−^-dependent Na^+^ transport.

Apples, as an important horticultural product, have significant economic benefits globally [39,40,41,42,43,44]. China has the highest apple production and nitrogen fertilizer application rates in the world. Excess nitrogen fertilizer is applied to cultivated apple trees to achieve high production, but the absorption efficiency of the plant body is less than 40%, which is a large discrepancy compared to developed countries [40,41,42,43,44] and causes serious environmental problems. Furthermore, the relatively poor local climate of apple orchards in China (mainly in mountainous, rural areas), insufficient rainfall, and reduced groundwater level, as well as soil salinization caused by unreasonable irrigation and single application chemical fertilizer over a long time period, mean that the apple trees are to a large extent vulnerable to drought and salt stress, resulting in a decline in apple yield and quality. Therefore, the potential role of *NRT1.1* in nitrate utilization and abiotic stress tolerance indicates the importance of identifying the NRT in apples [4,5,7,32,45].

In this study, a nitrate transporter protein, *NRT1.1,* was identified in apples, which promotes growth and development. We verified the expression response of *MdNRT1.1* to salt and drought stress, and we verified the function of *MdNRT1.1* under abiotic stresses. This study was designed as groundwork for future research on the mechanism of NRT1.1-mediated control of nitrogen nutrition and abiotic stress in apples.

## 2. Results

### 2.1. Phylogenetic Relationships and Multiple Sequence Alignment of MdNRT1.1

**Figure 1:**
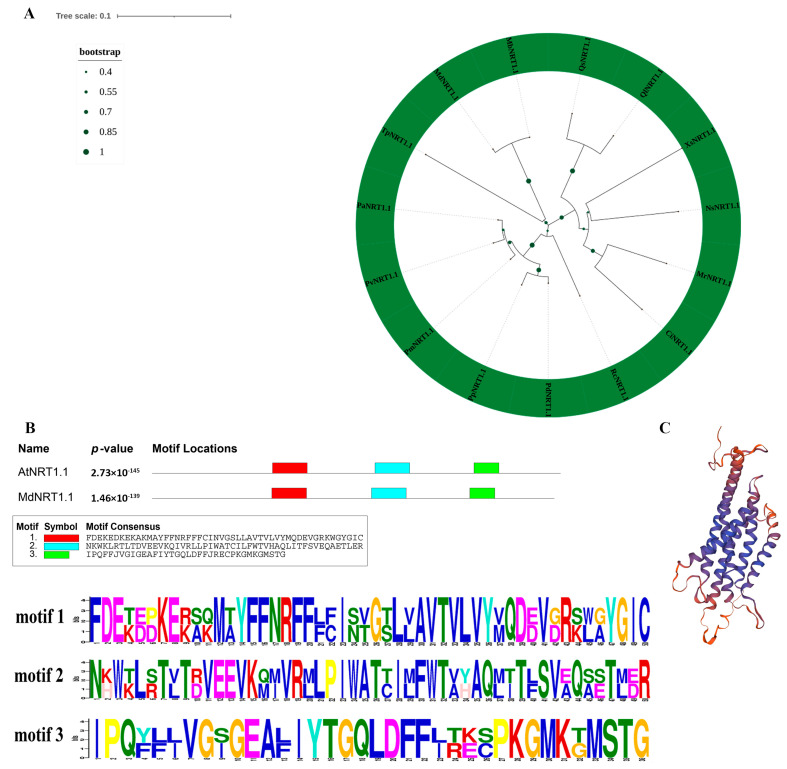
Phylogenetic tree analysis of *NRT1.1*. (**A**) Phylogenetic relationship analysis of MdNRT1.1 and 14 other plant NRT1.1 proteins obtained from the NCBI database. MdNRT1.1: *Malus domestica*, XP_008358135.1; CiNRT1.1: *Carya illinoinensis*, KAG6685326.1; MbNRT1.1: *Malus baccata*, TQE14061.1; MrNRT1.1: *Morella rubra,* KAB1217036.1; NsNRT1.1: *Nyssa sinensis*, KAA8522245.1; PaNRT1.1: *Prunus armeniaca*, CAB4279591.1; PvNRT1.1: *Prunus avium*, XP_021823895.1; PdNRT1.1: *Prunus dulcis*, XP_034214918.1; PmNRT1.1: *Prunus mume*, XP_008238567.1; PpNRT1.1: *Prunus persica*, ONI06660.1; QiNRT1.1: *Quercus Iobata*, XP_030948025.1; QsNRT1.1: *Quercus suber*, POE48521.1; RcNRT1.1: *Rosa chinensis*, XP_024168946.1; TpNRT1.1: *Trifolium pratense*, XP_045789551.1; XsNRT1.1: *Xanthoceras sorbifolium*, KAH7549611.1. (**B**) The conserved domains of *MdNRT1.1* and *AtNRT1.1* were analyzed by MEME. (**C**) The 3D structure map of the MdNRT1.1 protein.

The *MdNRT1.1* protein sequence was screened in the GDR (https://www.rosaceae.org/, accessed on 5 November 2021) using *AtNRT1.1* (AT1G12110) as the query. *MdNRT1.1* (MD15G1173800) was identified, and *NRT1.1* homologs from 15 species were identified to construct the evolutionary tree (Figure 1A). The data illustrated that apple *MdNRT1.1* had the closest genetic relationship with Mountain Wattles *MbNRT1.1* (Figure 1A), indicating that they diverged recently in evolution. All NRT protein sequences of the 15 species were highly similar and contained the PTR2 structural domain (Figure S5). Protein sequence analysis showed that MdNRT1.1 and AtNRT1.1 were extremely uniform in their model sites, while containing highly conserved structural domains (Figure 1B). In addition, the high-level structure of the MdNRT1.1 protein was predicted by the homology model (Figure 1C). This structure demonstrated that the secondary and tertiary structures of MdNRT1.1 are core conserved domains (Figure 1B,C).

The regulatory elements in the MdNRT1.1 promoter were predicted using PlantCARE. Among them, the GT1-motif is essential for the light response, the ARE regulatory element is essential for anaerobic induction, the MBS regulatory element is essential for the drought inducibility, the WUN-motif is essential for wound response, and the LTR regulatory element is involved in low-temperature responsiveness (Table 1). Moreover, some of the components associated with plant hormone responses have been predicted; in particular, the TCA element is involved in the salicylic acid response, and the GARE motif is involved in the gibberellin response (Table 1).

### 2.2. Nitrate and Abiotic Stresses Induce an Increased Transcript Level of MdNRT1.1

**Figure 2:**
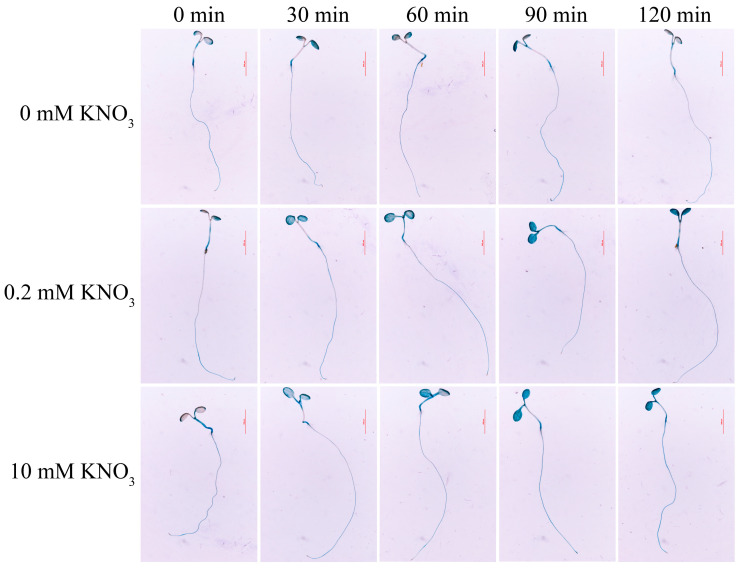
GUS staining in ProMdNRT1.1::GUS transgenic *Arabidopsis*. The ProMdNRT1.1::GUS transgenic *Arabidopsis* were treated with 2 mM KCl (as 0 mM KNO_3_), 0.2 mM KNO_3_, and 10 mM KNO_3_ for 0, 30, 60, 90, and 120 min.

To detect the expression of *MdNRT1.1* in response to nitrate, *ProMdNRT1.1::GUS* transgenic *Arabidopsis* was obtained and treated with different concentrations of nitrate, collected every 30 min before GUS staining. As shown in Figure 2, a high concentration of nitrate can lead to high expression of GUS activity with increasing time. The effect of GUS staining under a low concentration of nitrate treatment also increased with time, while no significant effect of GUS activity was found in 2 mM KCl (representing 0 mM NO_3_^−^) treatment. Meanwhile, Gala apples were treated with 2 mM KCl, 0.2 mM KNO_3_, and 10 mM KNO_3_, and samples were taken after 0, 1, 3, 6, 12, and 24 h of treatment. qRT-PCR was used to detect the transcript level of *MdNRT1.1* in response to different concentrations of nitrate (Figure S1A), which showed that the transcript level of MdNRT1.1 was increased by both nitrate treatments. MdNRT1.1 was also analyzed using GUS staining for spatiotemporal expression. The leaves and roots had the darkest staining status (Figure S2A). The expression of MdNRT1.1 in six different organ tissues (root, stem, leave, flower, fruit, and seed) of the Gala apple was also examined using qRT-PCR; the highest expression was in the roots and the lowest expression was in the seeds (Figure S2B). Overall, the above results suggested that *MdNRT1.1* is a nitrate-responsive gene and is expressed at different levels in different plant organs.

To investigate the expression of MdNRT1.1 in abiotic stress, we also used *ProMdNRT1.1::GUS* transgenic *Arabidopsis thaliana* for staining after different abiotic stress treatments. The GUS staining assay showed that the expression of MdNRT1.1 was upregulated under drought treatment, salt treatment, and ABA treatment in terms of the staining and GUS expression level (Figure S3). This result indicated that the expression of MdNRT1.1 was affected by drought stress, salt stress, and ABA.

### 2.3. Overexpression of MdNRT1.1 Affects Nitrate Utilization

**Figure 3:**
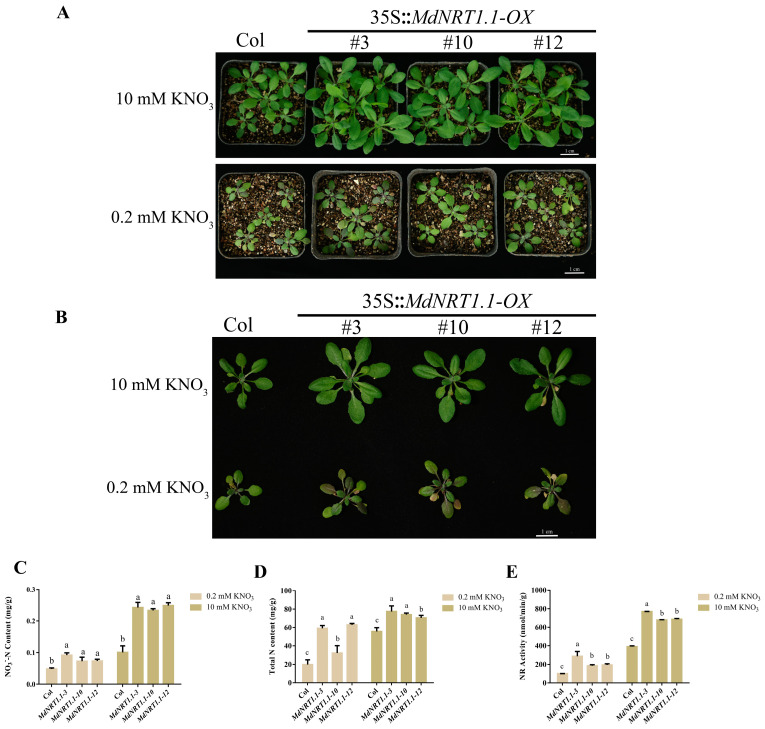
Overexpression of *MdNRT1.1* promotes the enlargement of adult Arabidopsis seedlings. (**A**) Col and three *MdNRT1.1 Arabidopsis* lines under high nitrate (10 mM) and low nitrate (0.2 mM) treatment. (**B**) Phenotypes of Arabidopsis plants after nitrate treatment. Statistical analysis of (**C**) NO_3_^−^-N content, (**D**) total nitrogen content, and (**E**) nitrate reductase (NR) activity in Col and transgenic lines after high/low nitrate treatments. Bars represent the mean ± SD (*n* = 3). Different letters above the bars indicate significant differences using the LSD test (*p* < 0.05).

To further explore the function of MdNRT1.1 in regulating nitrate use, MdNRT1.1 was ectopically transformed into Arabidopsis, and 3 overexpression lines with high expression were obtained from 12 overexpression lines (#3, #10, and #12) (Figure S3). Then, MdNRT1.1-OX and Col were treated in 1/2 MS medium containing 0.2 mM KNO_3_ or 10 mM KNO_3_ for 30 days (Figure 3A). Compared with Col, the MdNRT1.1-OX overexpression lines had a higher biomass (Figure 3A,B), following the higher NO_3_^−^-N and total nitrogen content (Figure 3C,D). MdNRT1.1 overexpression led to higher nitrate reductase (NR) activity (Figure 3E). These results suggested that MdNRT1.1 promoted nitrate assimilation and utilization and then promoted plant growth and development.

### 2.4. Overexpression of MdNRT1.1 Affects Root Development

**Figure 4:**
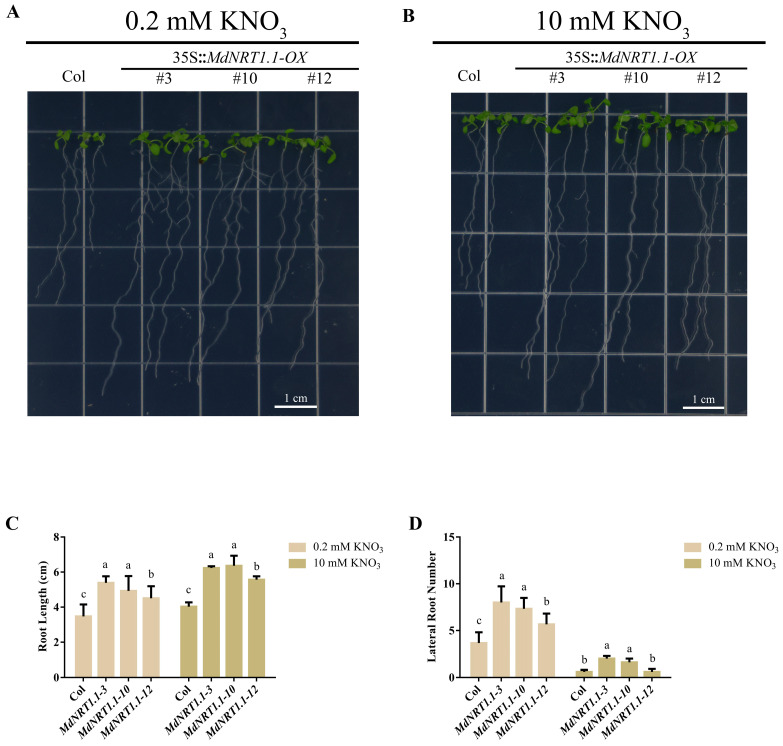
Effect of *MdNRT1.1-OX* in regulating root development. (**A**) Col and three *MdNRT1.1 Arabidopsis* lines on 1/2 MS medium supplement with 0.2 mM KNO_3_. (**B**) Col and three *MdNRT1.1 Arabidopsis* lines on 1/2 MS medium supplement with 10 mM KNO_3_. (**C**,**D**) Data related to the primary root lengths (**C**) and lateral root number (**D**) in Col and transgenic lines. Bars represent the mean ± SD (*n* = 3). Different letters above the bars indicate significant differences using the LSD test (*p* < 0.05).

To identify whether MdNRT1.1 has an impact on root development, MdNRT1.1-OX and Col were treated with different nitrate concentrations to detect root growth and development. Three days after germination, the Arabidopsis (MdNRT1.1-OX, Col) seedlings were transplanted into low NO_3_^−^ (0.2 mM KNO_3_) and high NO_3_^−^ (10 mM KNO_3_) medium. The results showed that transgenic Arabidopsis had longer primary roots than Col (Figure 4A–C). In addition, MdNRT1.1-OX had more lateral roots than Col, especially in the low nitrate treatment (Figure 4D). These results indicated that the overexpression of MdNRT1.1 promoted root development.

### 2.5. Overexpression of MdNRT1.1 Reduces Drought Tolerance

**Figure 5:**
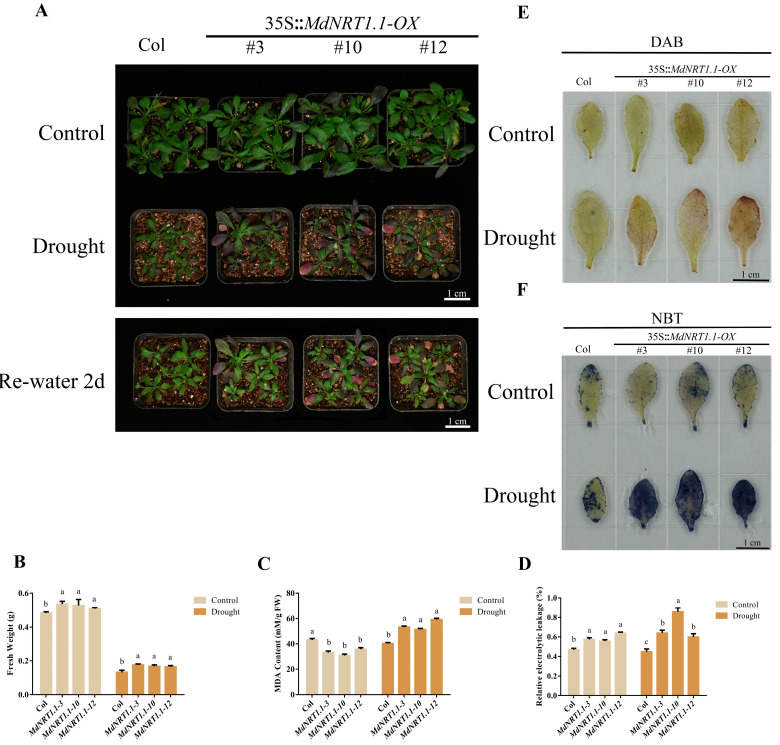
Overexpression of *MdNRT1.1* negatively regulates drought tolerance in plants. (**A**) Col and three *MdNRT1.1 Arabidopsis* lines under drought treatment. Statistical analysis of (**B**) fresh weight, (**C**) MDA content, and (**D**) relative electrolyte leakage in Col and transgenic lines under drought conditions. (**E**) Histochemical analysis of DAB staining of Col and three *MdNRT1.1* transgenics after drought treatment. (**F**) Histochemical analysis of NBT staining of Col and three *MdNRT1.1* transgenics after drought treatment. Bars represent the mean ± SD (*n* = 3). Different letters above the bars indicate significant differences using the LSD test (*p* < 0.05).

Expression analysis showed that an increased transcript level of MdNRT1.1 was induced by drought treatment (Figure S1B). To verify whether MdNRT1.1 was involved in drought stress tolerance, 7-day-old MdNRT1.1-OX and Col seedlings were treated in drought conditions for 30 days. The results showed that the biomass of MdNRT1.1 transgenic Arabidopsis was heavier than the control (Figure 5A,B), which may have been due to the fact that MdNRT1.1 absorbed more nutrients during the water-sufficient stage. The MDA content and relative electrolyte leakage of transgenic strains under drought treatment were higher than those of the wild type (Figure 5C,D). The results indicated that MdNRT1.1-OX was more sensitive to drought.

When plants are exposed to stress, intracellular reactive oxygen metabolism is disturbed and reactive oxygen species (ROS) accumulate. Hydrogen peroxide is the main ROS produced in plants, and staining samples using NBT and DAB can analyze the status of reactive oxygen species accumulation during abiotic stress in experimental materials. Before the drought treatment, there was no obvious difference between Col and MdNRT1.1 transgenic strains. However, after drought treatment, the MdNRT1.1 transgenic strain stained darker than Col. It was verified that MdNRT1.1 plants produce more ROS than Col (Figure 5E,F). In general, these results indicated that ROS accumulation in MdNRT1.1-OX plants was much higher than that in Col plants during drought conditions.

### 2.6. Overexpression of MdNRT1.1 Reduces Salt Stress Tolerance

**Figure 6:**
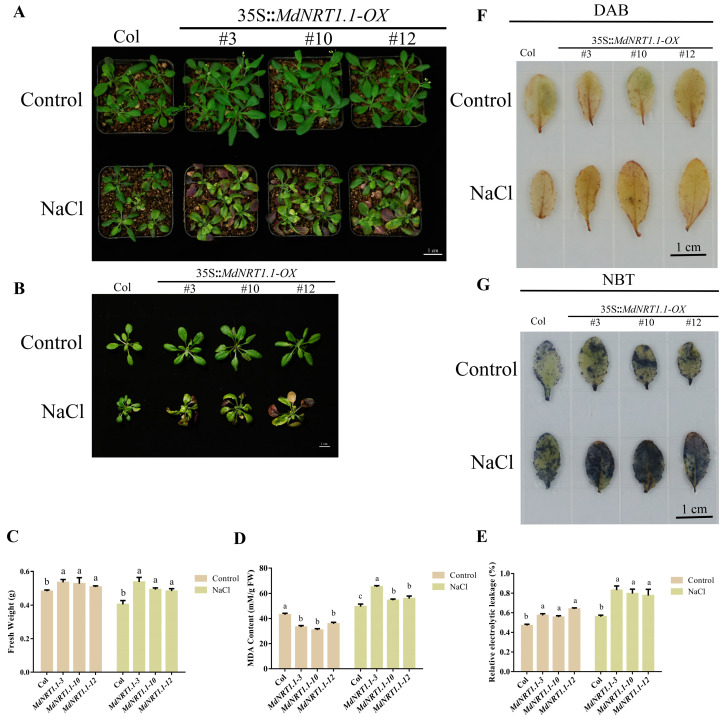
Overexpression of *MdNRT1.1* regulates salt stress tolerance. (**A**) Col and three *MdNRT1.1 Arabidopsis* lines under salt stress. **(B**) Phenotypes of single Arabidopsis plants after salt treatment. Statistical analysis of (**C**) fresh weight, (**D**) MDA content, and (**E**) relative electrolyte leakage in Col and transgenic lines after salt treats. (**F**) Histochemical analysis of DAB staining of Col and three *MdNRT1.1* transgenics after drought treatment. (**G**) Histochemical analysis of NBT staining of Col and three *MdNRT1.1* transgenics after drought treatment. Bars represent the mean ± SD (*n* = 3). Different letters above the bars indicate significant differences using the LSD test (*p* < 0.05).

Salt stress raised the transcription level of MdNRT1.1 (Figure S1C). Then, we proceeded to determine whether MdNRT1.1 was salt-resistant. After thirty days of high salt treatment, the biomass of transgenic MdNRT1.1 Arabidopsis thaliana was higher than that of the control (Figure 6A–C). The elevated MDA content and relative conductivity were obtained in the transgenic lines (Figure 6D,E). Relative conductivity and MDA concentration measurements demonstrated that MdNRT1.1 was intolerant to salt, while the effect of salt stress on the biomass of MdNRT1.1 plants was less.

The results of DAB and NBT staining demonstrated that the staining level of MdNRT1.1-OX lines was greater than that of Col, suggesting that the damage to the MdNRT1.1-OX lines under salt stress may have been more severe (Figure 6F,G). The results demonstrated that MdNRT1.1 enhances salt sensitivity.

### 2.7. Overexpression of MdNRT1.1 Changes Response to Exogenous ABA

**Figure 7:**
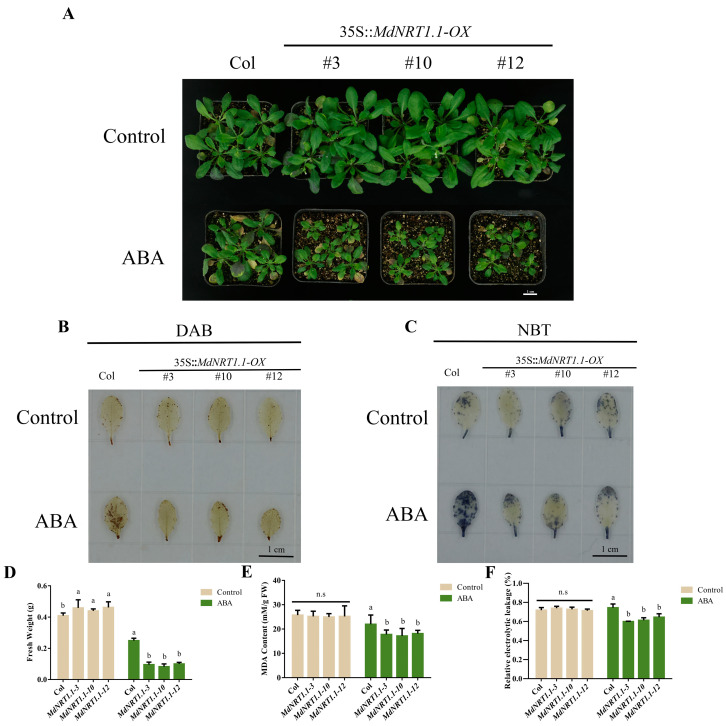
Overexpression of *MdNRT1.1* regulates ABA tolerance. (**A**) Col and three *MdNRT1.1 Arabidopsis* lines under ABA. (**B**) Histochemical analysis of DAB staining of Col and three *MdNRT1.1* transgenics after ABA treatment. (**C**) Histochemical analysis of NBT staining of Col and three *MdNRT1.1* transgenics after ABA treatment. Statistical analysis of (**D**) fresh weight, (**E**) MDA content, and (**F**) relative electrolyte leakage in Col and transgenic lines after ABA treatment. Bars represent the mean ± SD (*n* = 3). Different letters above the bars indicate significant differences using the LSD test (*p* < 0.05).

After ABA treatment, expression analysis revealed that the *MdNRT1.1* transcription levels rose (Figure S1D). Seven-day-old *Arabidopsis* plants were given ABA treatment for 30 days to verify if ABA had any impact on MdNRT1.1-OX plant development (Figure 7A,D). The plant size and fresh weight of the ABA-treated plants were smaller than those of the control, and the size and biomass of MdNRT1.1-OX plants in the ABA-treated group were considerably less than those of the Col plants (Figure 7A,D). The transgenic strain *MdNRT1.1* had considerably lower MDA levels and relative conductivity than Col based on the stated phenotype (Figure 7D–F). These findings suggested that ABA limited the growth of MdNRT1.1-overexpressing plants.

Before ABA treatment, neither DAB nor NBT staining of Col and *MdNRT1.1* transgenic lines revealed any significant changes (Figure 7B,C). After ABA treatment, staining of the *MdNRT1.1* overexpression lines revealed that the transgenic *MdNRT1.1-OX* lines staining region had a smaller surface area than Col (Figure 7B,C). These results indicated that ABA inhibited the growth of *MdNRT1.1* transgenic *Arabidopsis* but induces lower ROS generation in the plants. This scenario was consistent with the prior research that ABA inhibits the capacity of *MdNRT1.1* to absorb or transport nitrate, but more research is required [32].

## 3. Discussion

Nitrate is the most abundant inorganic nitrogen source in the soil. Its absorption, transport, and storage by plants are mainly accomplished by nitrate transport (NRT) proteins [4,5,32]. Research on *NRT1.1* gene-mediated nitrate uptake and transport in apples will be beneficial for improving nitrogen fertilizer utilization [5,6,39,45,46,47]. Moreover, recent studies have found that the nitrate transporter protein NRT1.1 plays an important role in various environmental stresses [32,37,48,49,50,51,52,53,54].

Previous studies have shown that *AtNRT1.1* was the first nitrate transporter found to affect nitrate uptake by plants [32]. NRT1.1 switches between high and low affinity via phosphorylation of Thr101 (T101) residue [36]. When ambient nitrate is scarce, *AtNRT1.1* is phosphorylated to dissociate the dimer and improve its flexibility, allowing it to function as a nitrate transporter with high affinity [9]. In this study, we cloned MdNRT1.1 based on the AtNRT1.1 protein sequence and investigated its role in nitrate utilization and drought and salt stress tolerance in apples.

To explore the nitrate response of *MdNRT1.1*, the heterologous expression of *NRT1.1* in *Arabidopsis* was considered (Figure S3). Previous studies have shown that NRT1.1 is a nitrate transporter protein and is abundantly expressed in the roots [32], and the results of GUS staining showed that MdNRT1.1 was also expressed in the highest amount in the roots. *MdNRT1.1* promoter construct also showed positive GUS staining under both high and low nitrate conditions (Figure 2). Considering that *MdNRT1.1* is a nitrate-responsive gene (Figure S1A), its expression is induced by NO_3_^−^. One study reported that under high nitrate conditions, *nrt1.1* mutants absorbed about 50% less nitrate than the wild type. When nitrate levels were below 0.25 mM, NRT1.1 was shown to have a 75% higher nitrate uptake in *Arabidopsis* than in the wild type [32,55]. Our evaluation of the role of *MdNRT1.1* in nitrate absorption revealed that the growth state, nitrate content, total nitrogen content, and nitrate reductase content of *MdNRT1.1-OX* plants were all greater than Col under both high and low nitrate conditions (Figure 3 and Figure 4).

Research shows that 50% of the annual yield loss of the world’s major crops is associated with abiotic stresses [56,57]. Recent studies have found that the nitrate signaling pathway provided by NRT1.1 also plays an important role in plant resistance to abiotic stress [32]. In the present experiment, the GUS staining assay indicated that the expression of MdNRT1.1 was upregulated in both staining and GUS expression levels under drought treatment (Figure S4). Transgenic *MdNRT1.1-OX Arabidopsis* plants were found to be less drought tolerant than wild-type plants under drought conditions (Figure 5), indicating that *MdNRT1.1* has an important role in plant responses and adaptation to drought stress. The accumulation of ROS was associated with the accumulation of MDA [58]. Under drought stress, overexpression of MdNRT1.1 led to higher MDA content and higher DAB and NBT staining levels, indicating more ROS production and greater tissue peroxidative damage in transgenic plants (Figure 5C,E,F). The increase in relative conductivity of MdNRT1.1 transgenic Arabidopsis under drought treatment also indicated that the membrane system was more severely damaged (Figure 5D). This was consistent with the findings of prior research showing *Arabidopsis* guard cells also express *NRT1.1*. The stomatal openings of *nrt1.1* mutants are smaller than those of wild-type plants grown in a nitrate-containing medium, resulting in improved drought tolerance. This may have resulted from a lack of *NRT1.1*, a decreased nitrate accumulation in guard cells, and the absence of nitrate-induced membrane depolarization [32,35].

In this research, *MdNRT1.1-OX* expression was found to be sensitive to salt stress treatments (Figure 6). In addition, the deepening of GUS staining and upregulation of the expression level GUS under salt stress treatment indicated that MdNRT1.1 reduced salt tolerance in plants (Figure S4). The MDA content of MdNRT1.1, the increase in relative conductivity, and the staining of DAB and NBT indicated that the plant tissues in MdNRT1.1-OX were severely damaged (Figure 6D–G), which may have been due to the fact that *NRT1.1* promoted the transport of Na^+^ from the roots to the shoots, leading to an attack of reactive oxygen species on the biological membranes in the plants, resulting in loss of membrane permeability function and disruption of photosynthesis and respiratory metabolism [37,38,48,49,50,51,52,53,54]. As salt stress mainly causes osmotic stress and peroxide accumulation in plants, and salt stress causes less damage to plants at the nutritional growth stage [59,60], the effect on fresh weight of MdNRT1.1 transgenic Arabidopsis may have been smaller (Figure 6C).

In this study, it was found that MdNRT1.1-OX plants were highly sensitive to drought and salt stress (Figure S1A,B). Several types of research have shown that ABA has an important relationship with plant resistance to abiotic stresses. ABA acts as a stress-responsive hormone that can induce a highly expressed number of resistant genes and physiological and biochemical adaptive responses [9,61]. This prompted us to explore the relationship between *MdNRT1.1* and exogenous ABA. By applying external ABA under normal conditions, we verified that transgenic *MdNRT1.1-OX* lines are highly sensitive to ABA and that the size and fresh weight of plants were significantly smaller than the wild-type plants (Figure 7A,D), but both NBT and DAB staining were lighter than the wild type. MDA content and conductivity were also similarly less than the wild type (Figure 7B,C,E,F), so ABA inhibited the growth of MdNRT1.1 transgenic Arabidopsis but induced a decrease in the production of ROS in plants, indicating that exogenous ABA may inhibit the transport of nitrate from the roots to shoots in apple seedlings [32].

## 4. Materials and Methods

### 4.1. Bioinformatics Analysis of the NRT1.1 Gene

Protein sequences of *NRT1.1* in *Arabidopsis* were obtained on the TAIR website, and the target protein database was searched through the blast tool on the GDR website (Apple Genome GDDH13 v1.1 proteins). The protein sequence of the apple gene was compared with the protein sequence in *Arabidopsis* based on BLASTp, and then we obtained the protein sequences of *NRT1.1* from the different species at the NCBI website via the protein sequence of apple *MdNRT1.1*. Utilizing these data, phylogenetic trees with reasonably close links were constructed.

The conserved protein domains of MdNRT1.1 were predicted by MEME (https://meme-suite.org/meme/tools/meme, accessed on 16 October 2022), the structure of the MdNRT1.1 protein was predicted by the SWISS-MODEL website (https://swissmodel.expasy.org/, accessed on 12 September 2022), and using the website of PlantCARE, we predicted cis-acting elements (http://bioinformatics.psb.ugent.be/webtools/plantcare/html/, accessed on 16 October 2022). The analysis of the conserved domains was performed on Clustal, where the first step was to enter the EBI web server through the Clustal Omega channel. The consequence was visualized and then modified from the Jalview (https://www.jalview.org/download/, accessed on 5 November 2021).

### 4.2. Plant Materials and Cultivation Methods

*Arabidopsis* Columbia (Col) and ten-year-old self-rooted “Gala” apple trees (Malus domestica “Royal Gala”), located in an apple orchard, were selected as the experimental materials of this study (Tai’an, Shandong, China) [62].

The tissues of apple samples, including the stem, root, leaf, flower, and fruit, were taken from a 10-year-old self-rooted apple tree to explore the gene expression pattern of apple trees.

*Arabidopsis thaliana* seeds were successively sanitized with 75% alcohol for 5 min and 1.5% sodium hypochlorite for 10 min and then cultured on 1/2 MS medium solid culture plates. The formulation of the culture medium contained 15 g L^−1^ sucrose and 8.0 g L^−1^ agar powder. Additionally, the pH value of the 1/2 MS media was adjusted to around 5.9 by 1.0 M sodium hydroxide, and then the seeds were vernalized at 4 °C for 3 d. These seeds were cultured and grown at 25 °C with a 16 h/8 h light/dark cycle [58].

The apple seeds were washed and dried on the exterior, and then the seeds were placed in a low-temperature (4 °C) incubation chamber for 3 months for vernalization. After germination, the seedlings were cultivated for four weeks in a 5 mM KNO_3_ nutrient solution, and then seedlings with constant growth status were transplanted into vermiculite under lengthy daylight circumstances (25 °C, 16 h/8 h light/dark) [62].

### 4.3. Nitrate and Abiotic Stress Treatments

For the nitrate treatments, similar-sized Gala seedlings were treated with low NO_3_^−^ (0.2 mM KNO_3_) or high NO_3_^−^ (10 mM KNO_3_) concentrations for 24 h under a 16 h/8 h light/dark photoperiod in an incubator at 25 °C. For nitrate treatment, seven-day-old *Arabidopsis thaliana* were transplanted to vermiculite and treated with low NO_3_^−^ (0.2 mM KNO_3_) or high NO_3_^−^ (10 mM KNO_3_) modified Hoglland nutrient solution at 10-day intervals [39].

For drought tolerance stress, similar-sized Gala seedlings were treated with 5% PEG6000 for 24 h under a 16 h/8 h light/dark photoperiod in an incubator at 25 °C. For drought treatment, seven-day-old *Arabidopsis thaliana* were transplanted into vermiculite and watered once, then watering was stopped.

For salt tolerance stress, similar-sized Gala seedlings were treated with 100 mM NaCl for 24 h under a 16 h/8 h light/dark photoperiod in an incubator at 25 °C. For salt treatment, seven-day-old *Arabidopsis thaliana* were transplanted into vermiculite and treated with 100 mM NaCl at 10-day intervals.

For ABA treatment, similar-sized Gala seedlings were treated with 30 μM ABA for 24 h at 25 °C under a 16 h/8 h light/dark photoperiod in an incubator. Seven-day-old *Arabidopsis thaliana* plants were transplanted into vermiculite and treated at 10-day intervals with 30 μM ABA.

### 4.4. Generation of Transgenic Materials

To obtain *MdNRT1.1-OX* material, the open reading frame (ORF) of MdNRT1.1 was inserted into the pRI-101 vector. A 2 kb segment upstream of the *MdNRT1.1* transcription initiation site was cloned and put into the pCAMBIA1300 vector to create *ProMdNRT1.1::GUS,* and the Agrobacterium tumefaciens LBA4404 strain was sustained on lysogeny broth (LB) medium with 50 mg/L of kanamycin and 50 mg/L of rifampicin. By using the floral dip transformation approach, the *MdNRT1.1* overexpression vector and *ProMdNRT1.1::GUS* constructs were introduced into *Arabidopsis thaliana* to produce transgenic *Arabidopsis* plants. Third-generation homozygous transgenic *Arabidopsis* was obtained (T3) (Shandong Agricultural University) [62]. The relevant primer information of the design is given in Table S1.

### 4.5. GUS Staining

The GUS staining buffer contained 1 mM 5-Bromo-4-chloro-3-indolyl-β-glutamic, 0.01 mM EDTA, 0.5 mM ferricyanide, 100 mM sodium phosphate (PH 7.0), and 0.1% (*v*/*v*) Triton X-100, and it was kept at 37 °C in the dark. A total of 2 mM KCl (representing 0 mM NO_3_^−^) and 0.2 mM and 10 mM NO_3_^−^ invaded the *Arabidopsis* at different time periods. The 7-day-old *ProMdNRT1.1::GUS* transgenic *Arabidopsis* GUS staining procedure was performed for 12 h, and then the transgenic *Arabidopsis* were immersed in GUS staining solution, followed by de-staining with anhydrous ethanol for 24 h [58].

The *ProMdNRT1.1::GUS* transgenic *Arabidopsis* was cultured with 2 mM potassium nitrate for 25 days, and the organs were stained with GUS for 12 h and then destained with anhydrous ethanol for 24 h.

### 4.6. Extracting Plant Genomic DNA and RNA

We used the Genomic DNA Reagent Kit and Omni Plant RNA Kit (tDNase I) to obtain plant DNA and RNA (Tiangen, Beijing, China) [39].

### 4.7. Real-Time Quantitative Polymerase Chain Reaction (qPCR)

We used the PrimeScript First Strand cDNA Synthesis Kit to synthesize the cDNA required for qPCR (Takara, Dalian, China). Real-time fluorescence quantitative analysis was used for qRT-PCR analysis with the UltraSYBR Mixture (Low Rox) kit (ComWin Biotech Co., Ltd., Beijing, China). The qRT-PCR experiments utilized the 2^−ΔΔCT^ method for analyses of the data [63].

### 4.8. Determination of Nitrate, Nitrate Reductase, Total Nitrogen, Electrolyte Leakage, MDA, and O_2_^−^

The samples were crushed into a powder, added to 1 mL of ddH_2_O, and then heated for 30 min at 100 °C. After 10 min of centrifugation at 12,000 rpm, the supernatant was collected in a flow cell. The nitrate concentration was determined with the AutoAnalyzer 3 continuous flow analytical instrument (SEAL Analytical, Mequon, WI, USA). The content of nitrate reductase and total nitrogen were identified using spectrophotometry through corresponding kits (Solarbio Life Science, Beijing, China).

The relevant treatment methods [58] were applied to detect electrolyte leakage. After grinding the experimental material well, we placed 0.1 g of the sample into a test tube and added deionized water to a volume of 10 mL. Extracts were soaked at room temperature for 12 h. The electrolyte leakage (R1) of the sample was measured using a relative conductivity meter. After this, the extract was heated in a boiling water bath for 30 min, and then the electrolyte leakage (R2) was measured. Relative electrolyte leakage = R1/R2 × 100%.

Using quartz sand and 2 mL of phosphate buffer, one gram of the sample was crushed into a homogenate. The homogenate was then combined with 5 mL of 0.5% thiobarbituric acid solution, heated for 10 min, and then centrifuged at 12,000 rpm for 10 min after chilling. At 450, 532, and 600 nm, absorbance values were measured to estimate the MDA content.

The accumulation of H_2_O_2_ and O_2_^−^ was detected by visualization of tissue localization staining of p-nitroblue tetrazolium chloride (NBT) and diaminobenzidine (DAB). The stained samples were of rosette leaves taken from the same location near the base. The leaves were immersed in DAB for 12 h, and the leaves were immersed in NBT solution for 6 h. Then, the leaves were decolored in absolute ethanol.

### 4.9. Data Analysis

All experiments were performed independently in triplicate except for those otherwise indicated. Values presented in this manuscript were expressed as means ± standard deviation (SD). The statistical significance of all data was determined using a one-way analysis of variance (ANOVA) and compared using Duncan’s test at the *p* < 0.05 level.

## 5. Conclusions

In conclusion, the expression of *MdNRT1.1* was affected by nitrate, which promoted root development in response to the nitrate environment, thus promoting nitrate uptake and transport. Overexpression of *MdNRT1.1* increased sensitivity to drought and salt stresses. In addition, the external application of ABA also limited the growth of *MdNRT1.1* transgenic *Arabidopsis*.

## Figures and Tables

**Table 1:** *Cis*-elements analysis of *MdNRT1.1* promoter regions.

*Cis*-Element Name	*Cis*-Element Sequence (5′-3′)	Function	Location
ARE	AAACCA	A *cis*-acting regulatory element essential for anaerobic induction	−15
MBS	CAACTG	MYB binding site involved in drought-inducibility	−1683
GT1-motif	GGTTAA	Light-responsive element	−591
WUN-motif	AAATTACT	Wound-responsive element	+1196
TCA-element	TCAGAAGAGG	*Cis*-acting element involved in salicylic acid responsiveness	+557
LTR	CCGAAA	*Cis*-acting element involved in low-temperature responsiveness	+1265
GARE-motif	TCTGTTG	Gibberellin-responsive element	−1905
CAT-box	GCCACT	*Cis*-acting regulatory element related to meristem expression	−791

## Data Availability

Not applicable.

## Supplementary Materials

The supporting information can be downloaded at https://www.mdpi.com/article/10.3390/ijms24119291/s1.
