# Supplementary material for: Identification and Functional Characterization of MdNRT1.1 in Nitrogen Utilization and Abiotic Stress Tolerance in Malus domestica
Source: Int J Mol Sci. 2023 May 26;24(11):9291. doi: 10.3390/ijms24119291 (PMC10252592; doi:10.3390/ijms24119291)
Supplement: Supplementary file 1 [file ijms-24-09291-s001.zip › ijms-2352116-supplementary.pdf]

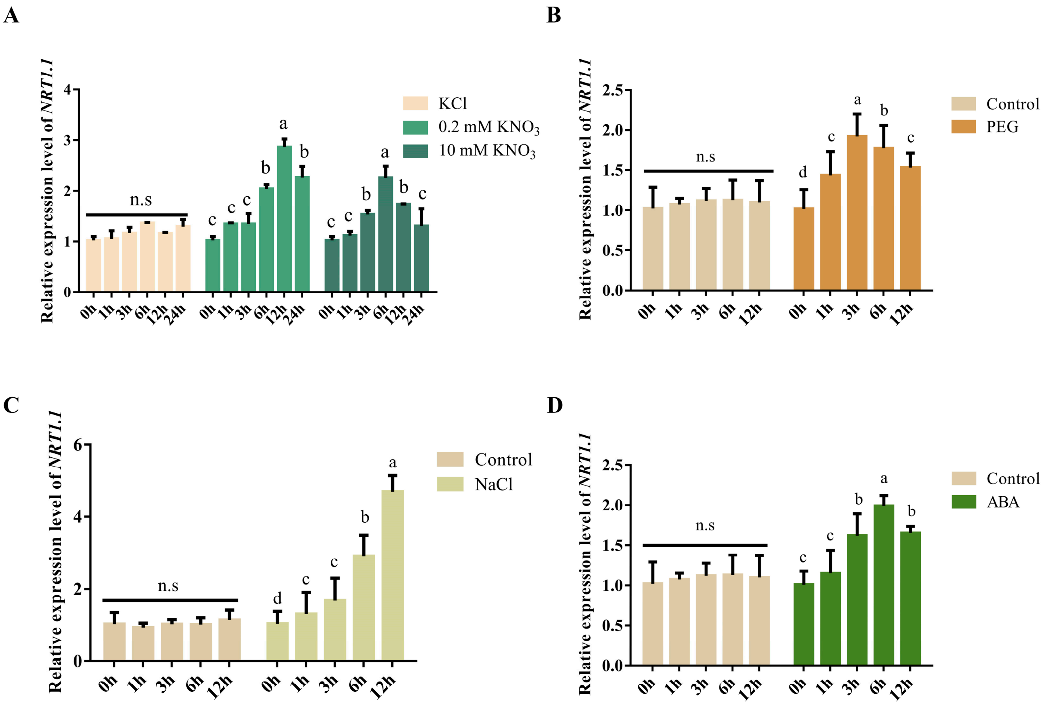

Figure S1. Expression patterns of the *MdNRT1.1* gene. (**A**) The expression patterns of *MdNRT1.1* genes under different nitrate concentrations (0.2 mM KNO_3 and 10 mM KNO_3, and KCl served as 0 mM KNO_3 control) for 0, 1, 3, 6, 12, and 24 h. (**B**) The expression patterns of *MdNRT1.1* genes under PEG treatment (to serve as drought stress) for 0, 3, 6, and 12 h. (**C**) The expression patterns of *MdNRT1.1* genes under NaCl treatment (to serve as salt stress) for 0, 3, 6, and 12 h. (**D**) The expression patterns of *MdNRT1.1* genes under ABA treatment for 0, 3, 6, and 12 h. Bars represent the mean ± SD (n = 3). Different letters above bars indicate significant differences using the LSD test ($p < 0.05$).

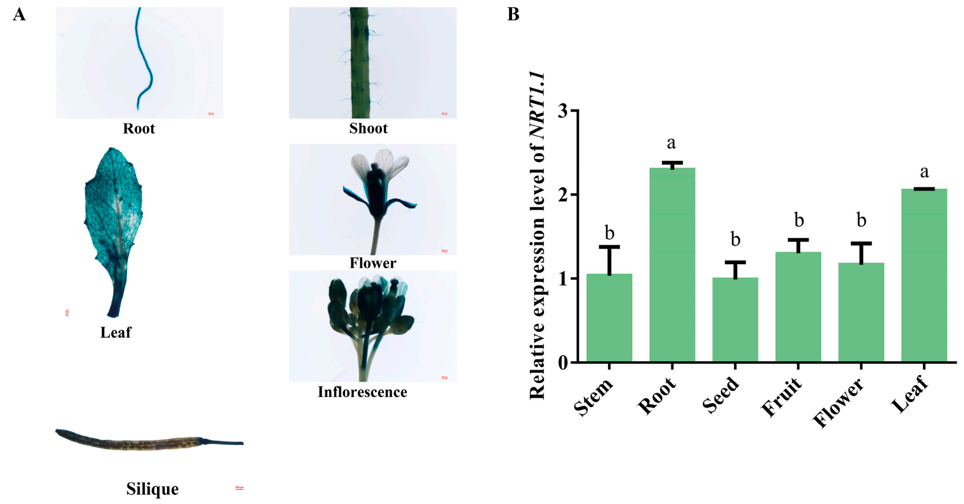

Figure S2. Tissue expression pattern of *MdNRT1.1*. (**A**) The GUS staining in *ProMdNRT1.1::GUS* transgenic under 2 mM KNO_3. (**B**) Organ (stems, roots, seeds, leaves, flowers, and fruits) expression analysis of *MdNRT1.1* by qRT-PCR in Gala. Bars represent the mean ± SD (n = 3). Different letters above bars indicate significant differences using the LSD test ($p < 0.05$).

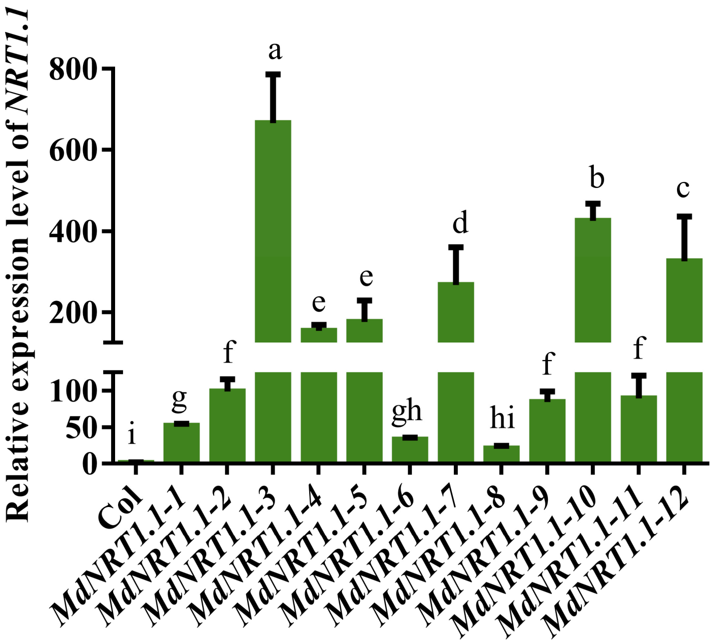

Figure S3. Detection of the transcript level of *MdNRT1.1* transgenic *Arabidopsis*. Bars represent the mean ± SD (n = 3). Different letters above the bars indicate significant differences using the LSD test ($p < 0.05$).

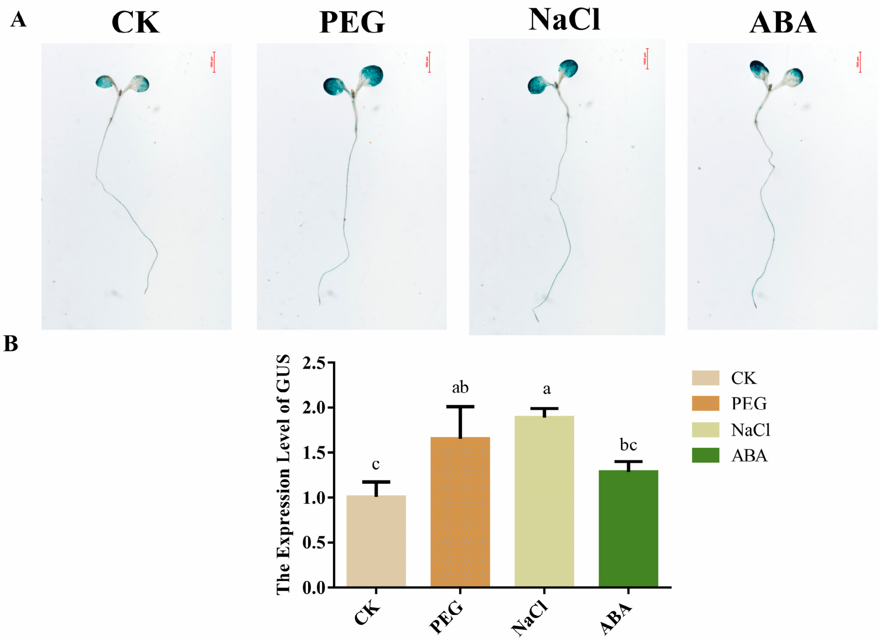

Figure S4. GUS staining in *ProMdNRT1.1::GUS* transgenic *Arabidopsis* in response to abiotic stresses. (**A**) The *ProMdNRT1.1::GUS* transgenic *Arabidopsis* was treated with 5% PEG, 100 mM NaCl, and 30 μM ABA, respectively. (**B**) The GUS expression of *MdNRT1.1* of (**A**). Bars represent the mean ± SD (n = 3). Different letters above the bars indicate significant differences using the LSD test ($p < 0.05$).

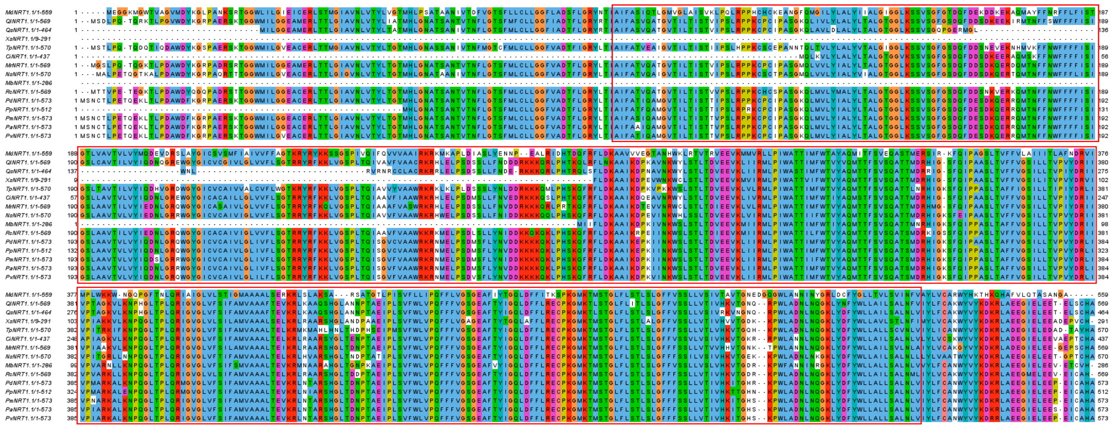

Figure S5. Alignment of amino acid sequences of MdNRT1.1 and other NRT1.1 proteins. The red box represents a conserved domain. They all had a Pfam domain.

Table S1. Primers for quantitative real-time PCR.

Primer name	Sequences (5′ to 3′)
MdNRT1.1-F (qRT)	5′-AGGCACAAGACAGTACCGTTTCAAG-3′
MdNRT1.1-R (qRT)	5′-CGTCAAGTCCGTTGGCAGTTCC-3′
MdNRT1.1-F (PRI)	5′-GGCGGCATGGAAGAAGAGGAAC-3′
MdNRT1.1-R (PRI)	5′-TGGTTGCTTGGGACACTGAGAATG-3′
MdNRT1.1-F (p1300-GN)	5′-AAGCTTGCATCTGACCATTTCTGGTT-3′
MdNRT1.1-R (p1300-GN)	5′-GGATCCTGTACTAACTAATGCCACTA-3′
Md18S-F (qRT)	5′-ACACGGGGAGGTAGTGACAA-3′
Md18S-R (qRT)	5′-CTCTTCTCAATCTCCCTGGATCG -3′
